# Color multilayer holographic near-eye augmented reality display

**DOI:** 10.1038/s41598-023-36128-x

**Published:** 2023-06-30

**Authors:** Alejandro Velez-Zea, John Fredy Barrera-Ramírez

**Affiliations:** Grupo de Óptica y Fotónica, Instituto de Física, Facultad de Ciencias Exactas y Naturales, Universidad de Antioquia UdeA, Calle 70 No. 52-21, Medellín, Colombia

**Keywords:** Optics and photonics, Displays

## Abstract

This study demonstrates a full-color near-eye holographic display capable of superimposing color virtual scenes with 2D, 3D, and multiple objects with extended depth upon a real scene, which also has the ability to present different 3D information depending on the focus of the user’s eyes using a single computer-generated hologram per color channel. Our setup makes use of a hologram generation method based on two-step propagation and the singular value decomposition of the Fresnel transform impulse response function to efficiently generate the holograms of the target scene. Then, we test our proposal by implementing a holographic display that makes use of a phase-only spatial light modulator and time-division multiplexing for color reproduction. We demonstrate the superior quality and computation speed of this approach compared with other hologram generation techniques with both numerical and experimental results.

## Introduction

Since its discovery by Dennis Gabor in 1948^1^, holography has become the cornerstone of many powerful techniques like metrology^2,3^, optical tweezers^4,5^, neural optostimulation^6,7^ and holographic displays^8,9^. This latest application has become the focus of renewed interest, as holographic displays could enable a true high-fidelity 3D visualization by reproducing the full phase and amplitude of a 3D scene. The full reproduction of the phase and amplitude can enable a holographic display to avoid the issues present in common implementations of virtual or augmented reality devices based on stereoscopy, such as the accommodation-vergence conflict^10^.

Most modern holographic display setups are designed to work in conjunction with computer-generated holograms (CGH) instead of actual experimental holographic recordings of real scenes. This eliminates the need for large and complex holographic recording setups and enables the generation of holograms from virtual objects. Despite this advantage, the calculation of holograms is a computationally intensive process^11^. A further challenge to the implementation of holographic displays is that the full complex modulation of a light field is difficult to achieve with current devices. This means that there is often a choice between phase-only or amplitude-only modulation, which places another constraint on the process of hologram generation. Furthermore, the limited resolution of current spatial light modulators (SLM) means that the quality of the reconstructed scenes is lower than desired for visualization applications^12^.

To deal with these challenges, many algorithms for computer-generated holography tailored to different scenarios have been proposed. One of the first such algorithms is the Gerchberg–Saxton (GS) algorithm^13^, an iterative method that enables the calculation of a phase connecting two different planes related by a Fourier transform. This approach can produce either phase-only or complex holograms; but it is limited to the reconstruction of 2D scenes, it is relatively slow, it is prone to stagnation, and the resulting holograms present significant speckle noise due the presence of random phase changes. Since the introduction of the GS algorithm, a large array of variations has been proposed, enabling the generation of Fresnel holograms^14^, the use of multiple constraints in each plane to improve convergence^15^, and more importantly, the generation of holograms of a single or multiple 3D scenes using a layer-based approach^16^.

Other iterative methods for hologram generation have been proposed, where the phase or amplitude is found solving an optimization problem with a well-defined loss function. Amongst these approaches we find both non-convex optimization algorithms^17,18^, gradient descent^19^, and as a more recent development, the use of deep learning^20,21^. Deep learning CGH algorithms have been particularly successful, leading to fast computation and very high-quality reconstructions. However, these algorithms still require a pre-computed training dataset, which must be generated using one of the previously mentioned algorithms and often lack generality, failing to generate holograms when the target scene differs significantly from those found in the training dataset.

Finally, there are CGH methods based on the use of point clouds or polygon decomposition^22,23^. These methods take advantage of the fact that simple geometric shapes have easily calculated diffraction patterns. These patterns can be pre-generated for different positions and stored in a lookup table (LUT)^24,25^. Then a target scene can be decomposed on a finite number of points, triangles, or lines and the diffraction pattern of each one retrieved from the lookup table, after which a hologram can be acquired by adding all resulting patterns. These methods can also result in high-quality holograms but require representing the target scene into large amounts of individual points or polygons, leading to slow computation. Another challenge is that, when the target scene is solid, occlusion calculations can further increase the computational complexity. Additionally, as the dimension of the scene increases, the size of the LUTs also increases rapidly, imposing large memory requirements on the computing hardware.

In this work, we are interested in the generation of color multiplane holograms of scenes containing multiple 3D and 2D objects placed in different axial depths, one behind the other, without crosstalk. This enables the reproduction of different scenes at different focus depths from a single hologram. This capability is desirable in varifocal displays, since a user can perceive different scenes depending on the focus plane of their eyes^26^. Beyond visualization, these kinds of holograms can also enable 3D control of light fields in extended volumes, a capability of that can allow the optoestimulation of complex neuronal distributions and the manipulation of several particles in 3D space using optical traps, to name some of the potential applications.

The algorithms used for generation of multiplane holograms are significantly more computationally intensive than those used to generate conventional holograms of 2D or 3D scenes. This is because in addition to encoding each scene into the hologram, they must also be optimized to avoid crosstalk between the information in each plane. The basic algorithms used to generate these holograms are the global^27^ and sequential^28^ Gerchberg–Saxton algorithms (GGS and SGS respectively). These are modifications of the basic GS, where all the individual planes of the multiple scenes to be incorporated into the hologram are optimized using alternative projections with the hologram plane. In particular, the GGS algorithm has demonstrated the capability for generating holograms with a significantly higher reconstruction quality than the SGS. However, both approaches require iteratively projecting each individual plane of the target scenes, making computation very slow^29^.

Other approaches used to generate multiplane holograms are the non-convex and gradient descent optimization, which presents better quality than the SGS and GGS but also shows limited computation speed^17,19^. To address this issue, we recently introduced a multiplane hologram generation method based on the singular value decomposition of the Fresnel transform function^30^. This approach makes light propagation computation planes significantly faster, which is a requirement of many iterative 3D hologram generation algorithms. Using this approach, we introduced a modification of the GGS called the two-step global iterative Fresnel algorithm (TGS-IFrTA), which leads to significantly faster hologram generation.

Despite these advances in multiplane hologram generation, there has been limited success in developing a full color multilayer holographic near-eye display capable of reproducing scenes where several objects can be presented at different distances from the user along the same axis without crosstalk. There are several challenges that must be considered to implement a near-eye display with this capability. First, there is the added computational time needed to generate a single-hologram per-color channel. Some recent works have applied modifications of the layer-based hologram generation to produce full-color reconstructions. For example, in the study by Yasuki et al*.*^31^, the authors implement a propagation approach based on the Hartley transform and real valued holograms to reduce the computation time for a full-color holographic scene in a factor of three. This offsets the increased computational cost of color hologram generation. Despite this, the scenes reproduced only contain a single 3D object, and thus no evaluation of the effect of crosstalk is demonstrated. Likewise, Zheng et al*.*^32^ introduced a compensation factor during hologram generation, improving the performance of the GGS. Then, they generate a multiplane hologram where the layers at different distances correspond to the color channel of a 2D object. This arrangement ensures that, at a desired distance, the correct superposition of color channels with each illumination wavelength is achieved. While this approach is capable of good quality reproduction of color 2D scenes, the need for different layers for each color channel limits the maximum axial extent of the objects or scene which can be reproduced with this technique.

From the viewpoint of near-eye displays, there are a broad range of contributions demonstrating the display of color scenes. For instance, Song et al.^33^ recently demonstrated a full-color near-eye display by generating a color hologram of a 2D scene using multiplexing encoding and a Maxwellian configuration. This method allows observers to view the same 2D reconstruction from the hologram at various focus planes, achieving a field of view of 32°. Similarly, Lin et al.^34^ implemented a binocular full-color near-eye display using frequency division multiplexing, where the frequency spectrum of the holograms for each color channel of a 3D scene were tailored to enable color display using a single SLM. Despite the effectiveness of these approaches, they are currently limited to displaying single 2D or 3D scenes. To address this limitation, techniques such as TGS-IFrTA are crucial for implementing a full-color near eye display that not only reduces computation time but also greatly reduces crosstalk in scenes with multiple objects. For this reason, implementing techniques like the TGS-IFrTA which not only decrease the computation time but also greatly reduce crosstalk in scenes with multiple objects are especially important for a full-color near eye display.

Secondly, the addition of multiple color channels makes the use of several coherent light sources that must be modulated independently with the corresponding multiplane hologram necessary. To achieve this, we must use several phase-only light modulators, which makes the system extremely complex, or take advantage of techniques like time-division^35^ or space-division multiplexing^36^. These techniques impose their own limitations on the framerate and resolution of the system and often require additional electronic control systems to function optimally. Finally, the holograms and optical elements of the near-eye multiplane holographic display must be implemented in such a way that the superposition of color channels is achieved accurately in a significant axial depth.

To address all these issues, we demonstrate a fully functional full-color holographic near-eye augmented reality, capable of presenting 2D, 3D, and multiplane information over a real scene in an extended axial volume without crosstalk while maintaining adequate color reproduction. In addition, we use our near-eye holographic display as an experimental test platform to demonstrate that the TGS-IFrTA is better suited to color multiplane hologram generation, offering improved quality and lower computation time compared with other conventional multiplane hologram generation methods. Numerical tests are also performed to quantify the performance of the hologram generation methods. The results of these tests are in agreement with the final experimental results.

## Results

### Color multiplane hologram generation methods

**Figure 1:**
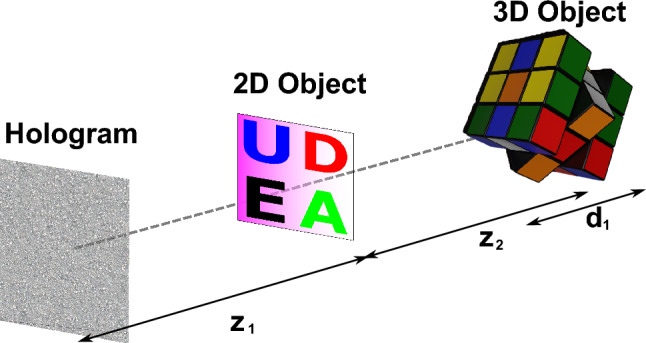
Example of a multiplane scene composed of a 2D and a 3D object.

For our tests, we will use a color scene composed of a 2D and a 3D object to highlight the flexibility of multiplane hologram generation. The desire is that both objects are reproduced at different distances from the hologram plane, as shown in Fig. 1.

**Figure 2:**
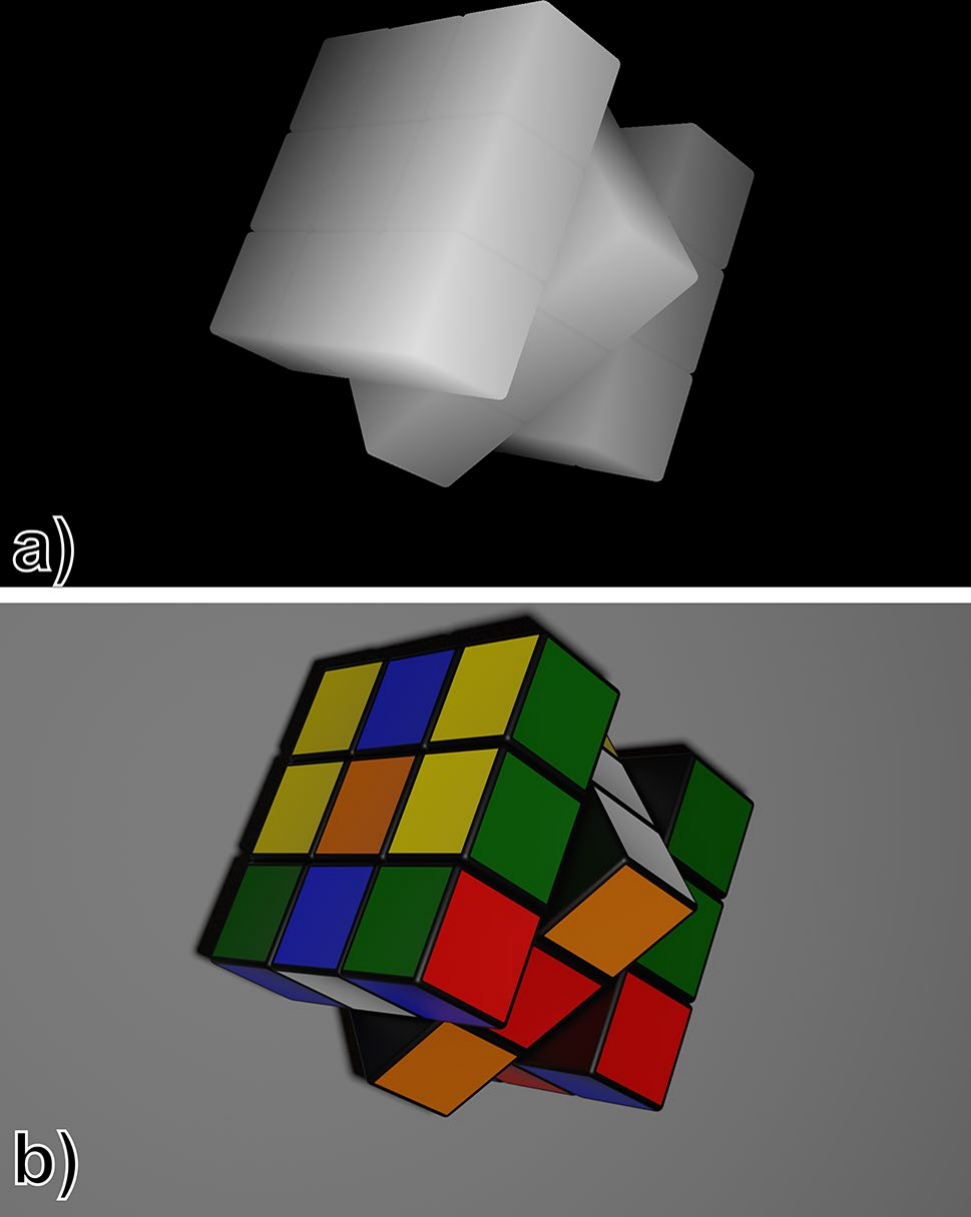
(**a**) Depth map of the 3D object. Darker colors indicate points at a further distance from the hologram plane, and lighter colors point closer to the hologram plane. (**b**) Intensity map of the 3D object. (“rubik cube” model by SDC performance licensed under CC-BY-4.0).

Here, the 2D object is at a distance of $z_{1}$ from the hologram plane, while the 3D object, with a axial length of $d_{1}$ is at a distance $z_{2}$ from the 2D object. Ideally, the requirement is that the final hologram reproduces only the 2D object at $z_{1}$ without any crosstalk or unfocused light corresponding to the 3D object. Likewise, the different planes of the 3D object found at $z_{1} + z_{2}$ should not present any crosstalk from the 2D object. First, to generate the hologram of the 3D object, we must decompose it into discrete layers. To achieve this, we use 3D modeling software to extract both an intensity and a depth map corresponding to the 3D object, as shown in Fig. 2.

The pixel value of the depth map represents the distance between the hologram plane and the corresponding point of the object. Taking advantage of this information, the 3D object can be discretized into a finite number of layers. In this case, 20 individual layers will be used.

Furthermore, color reproduction demands that we generate an individual hologram for each color channel. Three color channels will be used, corresponding to the red, green, and blue colors. This means that, to reproduce the full-color scene of Fig. 1, there is a total of 63 individual planes which must be codified into three holograms; this corresponds to the three-color channels of the 2D object and 20 planes for each color channel of the 3D object. This large number of planes is precisely what makes color multiplane holograms challenging to compute. To test the effectiveness of the method proposed in this study, two standard hologram generation methods for comparison will first be introduced along with the way a scene composed of multiple color 3D scenes can be generated with those methods.

### Iterative Fresnel algorithm layer multiplexing

**Figure 3:**
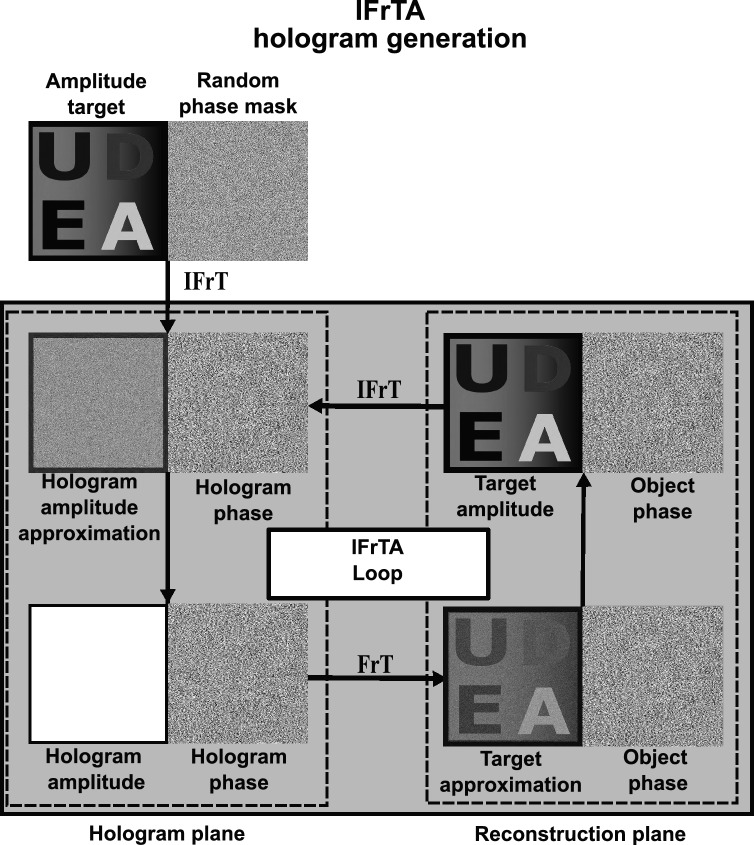
Flowchart of the IFrTA phase hologram generation technique. FrT: Fresnel transform, IFrT: inverse Fresnel transform.

One way to generate a hologram of a 3D scene is by generating the individual holograms of each layer and then multiplexing all those holograms together. To generate the hologram of each layer, we use the iterative Fresnel algorithm (IFrTA) shown in Fig. 3.

In this algorithm, we first multiply a target amplitude with an initial random phase. Then, we backpropagate the result to the hologram plane using an inverse Fresnel transform (IFrT). In the hologram plane, the amplitude of the resulting field is replaced with a constant. We then propagate the result to the target layer using a Fresnel transform (FrT) and replace the resulting amplitude with the target amplitude. This procedure is repeated; and in each iteration, the amplitude obtained in the target layer will be a closer approximation to the target. After a set number of iterations or when the amplitude in the reconstruction plane is a good approximation of the target, as given by some quality metric, the phase in the hologram plane can be taken as the final hologram.

To generate the multiplane hologram of Fig. 1, we apply the IFrTA to each individual layer of each color channel, obtaining 21 holograms per color channel. Then, we add together all the holograms of a single channel and set the amplitude of the result as a constant. The resulting phase will be the multiplane hologram for that color channel. This process is then repeated for the remaining two-color channels, allowing us to generate holograms for the full-color representation of the scene.

From the description of this approach, we can see that this method, while straightforward, has large computational requirements. Each iteration of the IFrTA needs two Fresnel transforms (a direct and an inverse one), and to obtain a reasonable approximation to the amplitude of each layer at least 20 iterations are desirable. This leads to a total of $3\times N\times 2\times 20$ Fresnel transform computations needed to obtain the holograms of the 3 color channels, where *N* is the number of layers to be computed (21 for the scene of Fig. 1). As we will show, this method is ill-suited for multiplane holograms, since there is no optimization step that takes account of all planes as a whole. This leads to severe crosstalk, highlighting the need for more sophisticated methods.

### Global Gerchberg–Saxton algorithm

**Figure 4:**
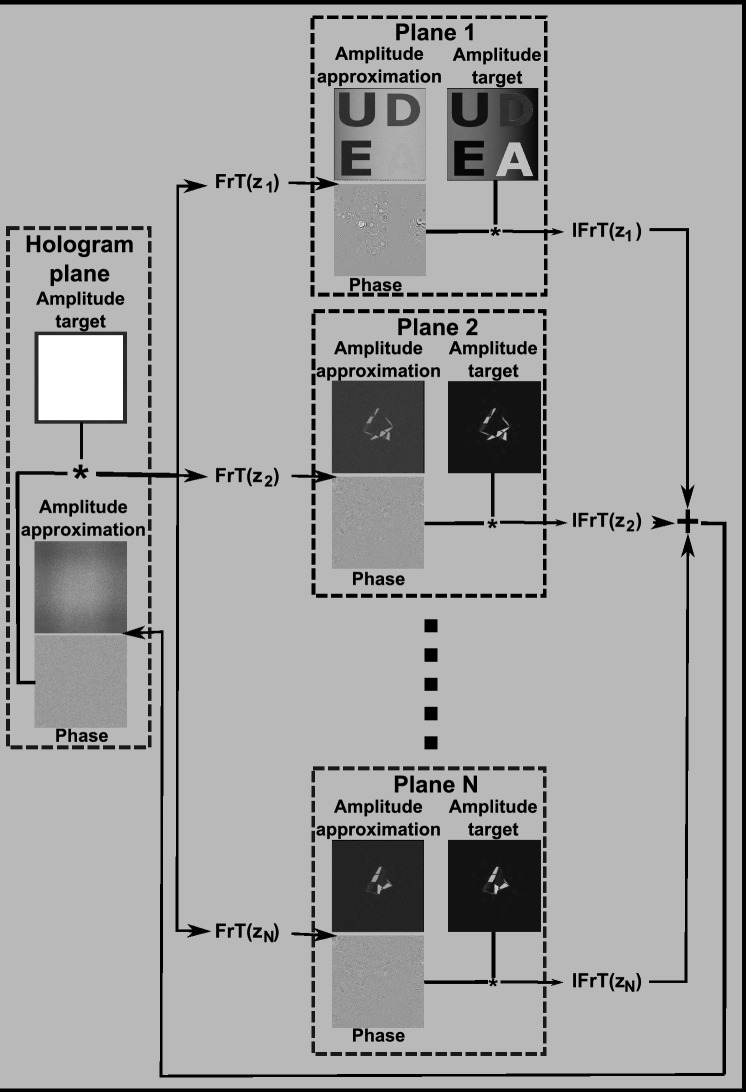
Flowchart of the GGS hologram generation method. ${z}_{1}$, ${z}_{2}$…${z}_{N}$: distance between the layer 1, 2, … N and the hologram plane.

A second approach for multiplane hologram generation is the GGS. In this method, the same 21 layers are taken per channel of the scene in Fig. 1 and apply a modified G–S algorithm, as shown in Fig. 4.

The GGS has some similarities to the IFrTA layer multiplexing method. The main difference is that, instead of adding the holograms of all layers after the full IFrTA iterations have been completed, we first propagate the field in the hologram plane to each individual layer. Then, we apply the amplitude constraint, replacing the amplitude in every layer with the target amplitude, and back propagate the result to the hologram plane. Afterwards, the resulting field from each layer is added together, and the amplitude is set to a constant. This process is repeated iteratively a set number of times or until some quality threshold is reached.

The main advantage of the GGS over the IFrTA layer multiplexing is that the phase in the hologram plane is optimized in each iteration considering the contribution of all individual layers of the scene. This reduces crosstalk between planes and significantly increases the reconstruction quality. On the other hand, the computation time of both methods is nearly identical. Here, we also require an FrT and an IFrT per layer per iteration, leading to the same total number of $3\times N\times 2\times 20$ Fresnel transformations to obtain the holograms of three-color channels.

## Two-step global iterative Fresnel algorithm

Finally, we will introduce our proposed method. As we have seen in both the IFrTA layer multiplexing and the GGS^27^, to obtain the holograms of the three-color channels in a scene, we require at least $3\times N\times 2\times 20$ Fresnel transformations. To calculate the Fresnel transform between two planes, one of the most common and efficient approaches is the angular spectrum (AS) method^37^, where FrT of an initial field $g(x,y)$ at a distance $z$ is given by

1 $$FrT_{z} \left\{ {g(x,y)} \right\} = F^{ - 1} \left\{ {F\left\{ {g(x,y)} \right\}F\left\{ {h_{z} (x,y)} \right\}} \right\}$$

where the operators $F\{ \}$ and $F^{ - 1} \{ \}$ represent the Fourier transform (FT) and the inverse Fourier transform (IFT), and $h_{z} (x,y)$ is the Fresnel impulse response (IR) function for a distance $z$, given by

2 $$h_{z} (x,y) = \frac{1}{j\lambda z}\exp \left[ {\frac{jk}{{2z}}\left( {x^{2} + y^{2} } \right)} \right]$$

where $\lambda$ is the illumination wavelength.

Usually, the computation of Eq. 1 can be performed relatively fast using a fast Fourier transform algorithm. However, as the number of layers and the resolution of each layer increases, the amount of Fresnel transforms required makes hologram calculation difficult.

A way to make this calculation easier is to make use of the singular value decomposition of the Fresnel IR function. As we can see from Eq. 2, this function is separable, which means that it only has a single singular value. As such, if we can write the Fresnel IR as a matrix with size $[M\times N]$, we can then decompose it as.

3 $$h_{z} [n,m] = U[n]S[n,m]V[m],$$

where $U$ and $V$ are vectors with size $[1 \times N]$ and $[M \times 1]$, and $S$ is a matrix with size $[M \times N]$ with a single non-zero value that corresponds to the single singular value of $h_{z} [n,m]$. In our case, this singular value is unity.

This way, we can perform the calculation of the Fresnel transform of a given discrete field $g[n,m]$ as two one-dimensional convolutions, given by.

4 $$FrT_{z} \{ g[n,m]\} = \{ g[n,m] \otimes U[n]\} \otimes V[m].$$

where ⊗ denotes a one-dimensional convolution operation. This is approach is also known as separable convolution, and is often used in point based hologram generation to significantly speed up hologram computation^38,39^. However, the SVD formalism can also be used even if the function is not separable, which can happen in propagation involving optical aberration corrections^40^. If the size of the Fresnel impulse response is a small vector, the calculation of the two one-dimensional convolutions of Eq. 4 can be faster than the angular spectrum approach of Eq. 1. However, the Fresnel IR has an infinite extent; and for computational calculations, it is taken as the same size as the input field $g[n,m]$. To solve this issue, we demonstrated in a previous work that it is possible to perform an approximated propagation calculation using a truncated Fresnel IR. We will refer to the propagation computation using Eq. 4 and a truncated Fresnel IR as convolution with singular value decomposition (C-SVD). In particular, the error in the propagation introduced by truncating the Fresnel IR becomes smaller as the propagation distance decreases. This way, propagation using Eq. 4 and a truncated Fresnel IR can be more efficient than the conventional angular spectrum method when large amounts of Fresnel transforms between planes with small separations must be calculated.

**Figure 5:**
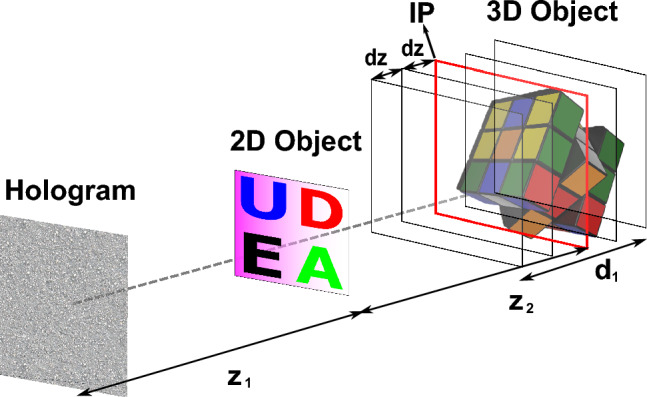
Layer division in the TSG-IFrTA algorithm. IP: intermediate plane, ${z}_{1}$, ${z}_{2}$: distances between objects, ${d}_{1}$: axial length of the 3D object, $dz$: distance between layers.

To take advantage of this capability, we introduced the two-step global iterative Fresnel algorithm (TSG-IFrTA). In this algorithm, we perform the same iterative procedure as in the GGS. However, the light between the different planes in the target scene is propagated using the truncated Fresnel IR to intermediate planes, chosen in such a way that the distance between a given layer and the closest intermediate plane is small. This is a similar approach to the wavefront recording planes^38^ originally introduced in point cloud hologram generation. However, in our case, it is applied to a layer-based iterative approach. The use of these intermediate planes ensures that a small size Fresnel IR can be used for this propagation without significant loss of accuracy due to truncation, leading to a significant increase in computation speed. For longer propagations between intermediate planes, we then make use of the angular spectrum method. An example of this scheme applied to the scene of Fig. 1 can be seen in Fig. 5.

In Fig. 5, we can see that an intermediate plane is defined in the middle of the 3D object. Then, each of the different layers of this object must be close enough to this intermediate plane to ensure that propagation to the intermediate plane using the C-SVD is faster than direct propagation using the angular spectrum method. If the number of layers of the object is increased, since its axial length remains constant, the distance dz between layers will decrease, and smaller truncated Fresnel IRs will become possible, further decreasing the computation time. If the axial length is greater than the necessary minimum to ensure the effectiveness of the truncated Fresnel IR when propagating to the intermediate plane, additional intermediate planes can be defined.

**Figure 6:**
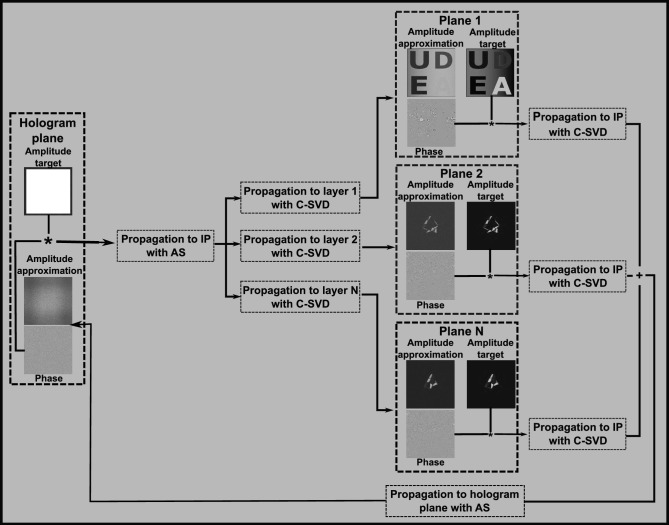
Flowchart of the TSG-IFrTA for multiplane hologram generation with a single intermediate plane.

Figure 6 shows the flowchart of the TSG-IFrTA for the case of a single intermediate plane. If there are additional intermediate planes, the fields from each must be backpropagated to the hologram plane and added together before applying the hologram plane constraint.

The TSG-IFrTA means that the computation time for multiplane hologram generation is no longer directly proportional to the number of propagations since shorter propagations can be calculated faster. As such, if we increase the number of layers of a given object without increasing its dimensions, the distance between layers will decrease, compensating the existence of additional layers with the capability for faster propagation by means of the truncated Fresnel IR.

### Numerical results

**Figure 7:**
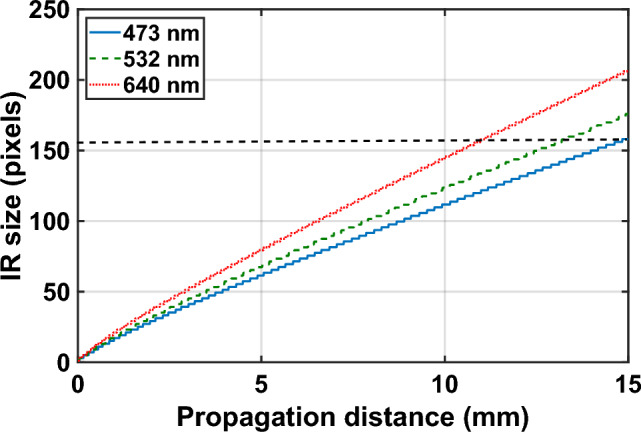
Truncated IR size in pixels vs propagation distance. The black dashed line marks the IR Fresnel size where the time for propagation computation using C-SVD is the same as the conventional AS approach. This size is equal to 160 pixels.

Before evaluating each of the hologram generation methods detailed in the previous section, it is important to analyze the propagation distances required to ensure that the TGS-IFrTA provides optimal performance for all wavelengths involved in our color hologram generation. As demonstrated in our previous work^30^, we truncate the Fresnel impulse response (IR) to a size corresponding to the area where 99.9% of its energy is found. This area, and therefore the Fresnel IR size in pixels, increases with longer propagation distances. Moreover, the Fresnel IR size also depends on the light wavelength, with shorter wavelengths resulting in smaller Fresnel IR for the same propagation distance. Figure 7 illustrates how the truncated Fresnel IR size varies with propagation distance for three wavelengths (473 nm, 532 nm, and 640 nm), which correspond to the blue, green, and red channels for color hologram generation.

Based on the results shown in Fig. 7, it can be observed that the maximum propagation distance required to ensure that the C-SVD propagation with truncated Fresnel IR is faster than the AS method is 14.7 mm for 473 nm, 13.7 mm for 532 nm, and 11.45 mm for 640 nm. In practical terms, this implies that the distance between each plane of the scene and the closest intermediate plane should be kept below 11.45 mm to ensure that the advantage in computation time of the SVD method is maintained for all wavelengths.

We now test the accuracy of the C-SVD propagation method with a truncated Fresnel IR. To achieve this, we calculate a 2 mm, 10 mm, and 50 mm propagations using both the conventional AS method and the C-SVD with a truncated Fresnel IR for a rect function of size $100\times 100$ pixels, using a space with a total size of $1920\times 1920$ pixels. The pixel size was set at 8 µm and the illumination wavelength at 532 nm. The computation time and the correlation coefficient between the truncated Fresnel IR result and the angular spectrum one was then calculated.

**Figure 8:**
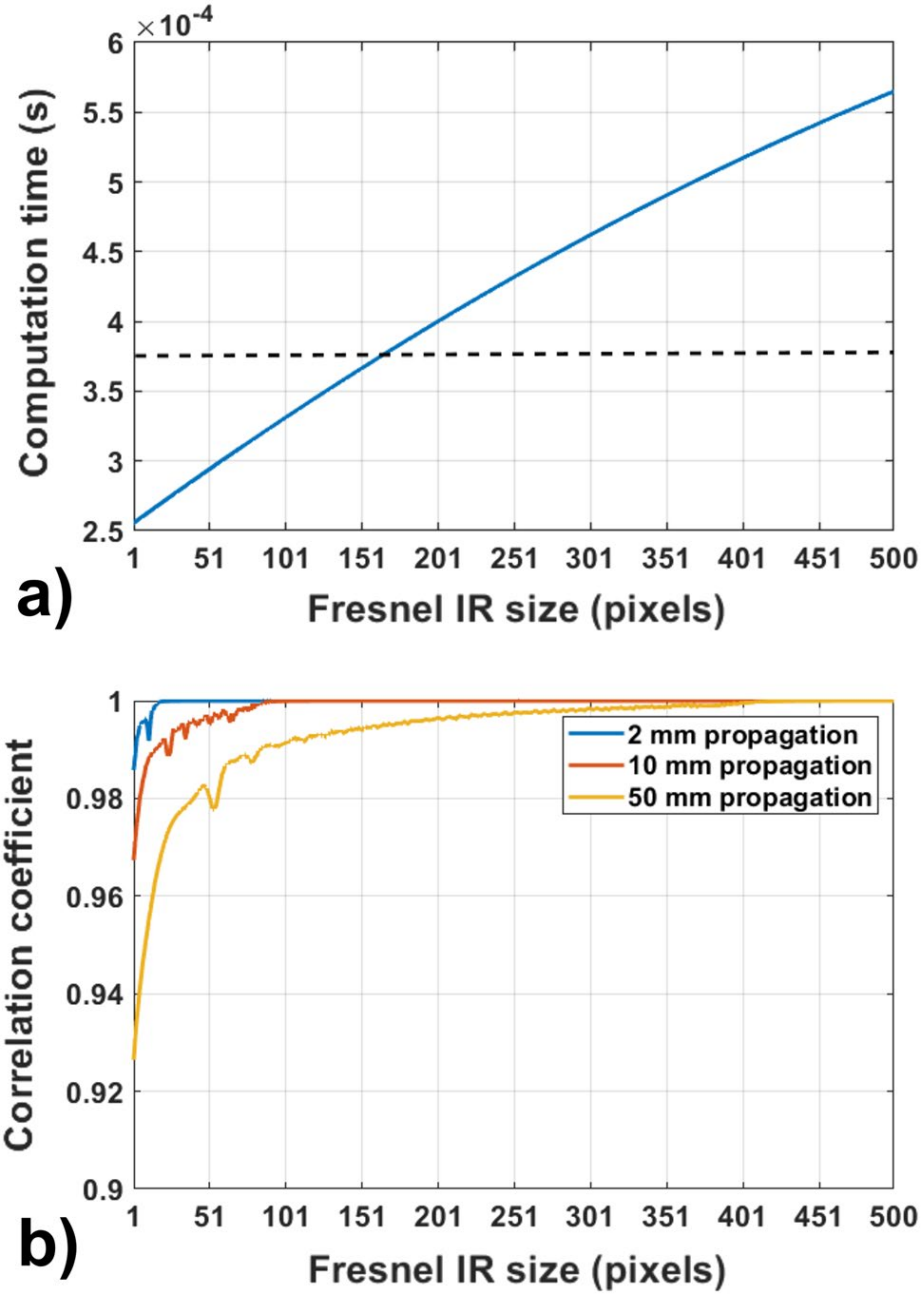
(**a**) Solid line: computation time for the C-SVD propagation vs size of the Fresnel IR, dashed line: computation time with AS. (**b**) Correlation coefficient between the intensity after propagation with C-SVD and AS vs size of the Fresnel IR.

As can be seen from the results of Fig. 8a, the computation time for propagation with C-SVD falls significantly as the size of the truncated Fresnel IR becomes smaller. In particular, notice the dashed line in Fig. 8a, which represents the time needed to perform the propagation using the AS method. This time is nearly 0.37 ms, and with a Fresnel IR with a size smaller than $160\times 160$, we can perform propagation with the C-SVD approach faster. From the results of Fig. 7b, we can see that for a propagation with a distance of 2 mm, a Fresnel IR with the size of $20\times 20$ can be used while maintaining a correlation coefficient of nearly 1. This leads to a computation time of nearly 0.26 ms compared to the 0.37 ms of the AS method. To maintain high accuracy for 10 mm and 50 mm propagations, Fresnel IR sizes of $90\times 90$ and $410\times 410$, respectively, are necessary. This corresponds to a computation time of 0.33 ms for 10 mm and 0.52 ms for 50 mm. Based on the results shown in Figs. 7 and 8, it can be concluded that Fresnel IRs with sizes of 21, 20, and 19 pixels can be used for 473 nm, 532 nm, and 640 nm wavelengths, respectively, when the propagation distance is 1 mm. These choices result in a computation time of approximately 0.26 ms for all three wavelengths.

We now proceed to test and compare each of the hologram generation methods proposed in Sect. 2. In this case, we generate the hologram of each color channel of the scene of Fig. 1 using the IFrTA multiplexing, the GGS, and the TSG-IFrTA methods. The total computation time needed to generate the holograms of the three-color channels was 12.367 s for the IFrTA multiplexing, 11.508 s for the GGS, and 5.198 s for the TSG-IFrTA.

**Figure 9:**
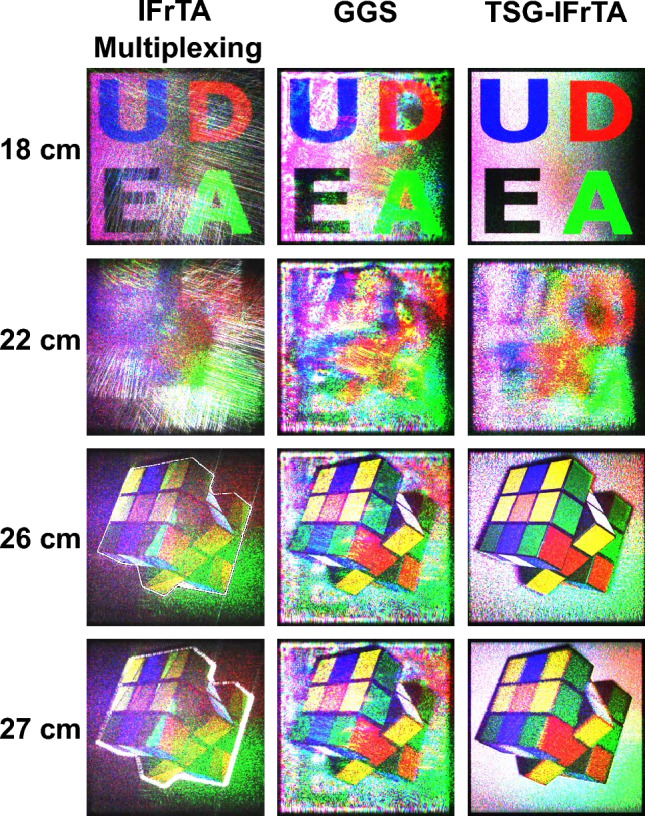
Numerical reconstruction results for color holograms generated with the IFrTA multiplexing, the GGS, and the TSG-IFrTA.

Once the holograms are generated, we perform their numerical reconstruction by propagating the field from the hologram plane to distances ranging from 16 to 30 cm. The results of these reconstructions for selected planes are shown in Fig. 9. In the result at 18 cm, we see the 2D object in focus. The result at 22 cm is an intermediate plane between both objects, the result at 26 corresponds to an intermediate plane of the 3D object in focus, and the result at 27 cm is the back plane of the 3D object in focus. Videos demonstrating the numerical reconstruction of the holograms in all planes from 16 to 30 cm are shown in Visualization 1 for the IFrTA multiplexing, in Visualization 2 for the GGS, and in Visualization 3 for the TSG-IFrTA. We can see that the TSG-IFrTA has a significant reconstruction quality advantage with no noticeable crosstalk or artifacts in either object. While the GGS also reconstructs both the 3D and the 2D object, there are noticeable artifacts. Finally, the IFrTA multiplexing provides very deficient reconstruction of both objects, in particular for the 3D object. The presence of strong diffraction in the borders of the layers of the 3D object in this last case causes a very noticeable loss of quality. This is due to the lack of iterative optimization of the hologram plane constraint, which is only applied once in this technique.

To quantitatively measure the difference in reconstruction quality for the holograms obtained with each method, we calculated the correlation coefficient (CC) between two reference images of the 2D and 3D objects of the target scene and the intensity obtained after numerical reconstruction at different planes.

**Figure 10:**
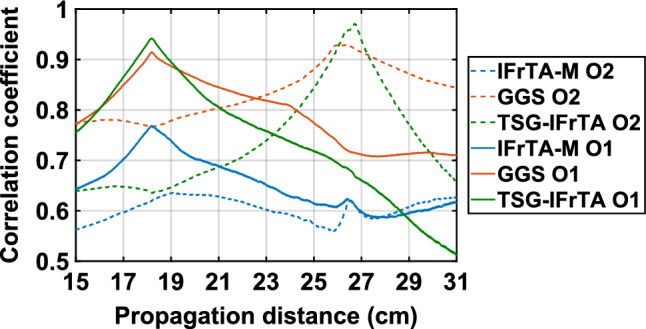
Correlation coefficient between an in-focus image of the 2D object (O1), the 3D object (O2), and different reconstruction planes obtained from holograms generated with IFrTA multiplexing, GGS, and TSG-IFrTA.

From the results of Fig. 10, we can confirm that the TSG-IFrTA has the best correlation coefficient for the reconstruction of both objects and the IFrTA multiplexing the worst. We can also notice how there is no peak for the CC at 26 cm in the case of the IFrTA multiplexing, which is caused due to the strong diffraction effect between the layers of the 3D objects previously discussed.

Finally, we will evaluate the performance of all hologram generation methods described in this paper for different distances between the objects of our test scene. To accomplish this, we placed both objects with a distance of 1 mm, 10 mm, 15 mm, 25 mm, and 50 mm between them, respectively. Then we generated the corresponding holograms using IFrTA multiplexing, GGS, and TSG-IFrTA. Finally, we reconstruct the scene and display the planes of best focus of both objects. It is worth noting, in this case, that the distances between objects are measured between their closest planes, not between the center of each object.

**Figure 11:**
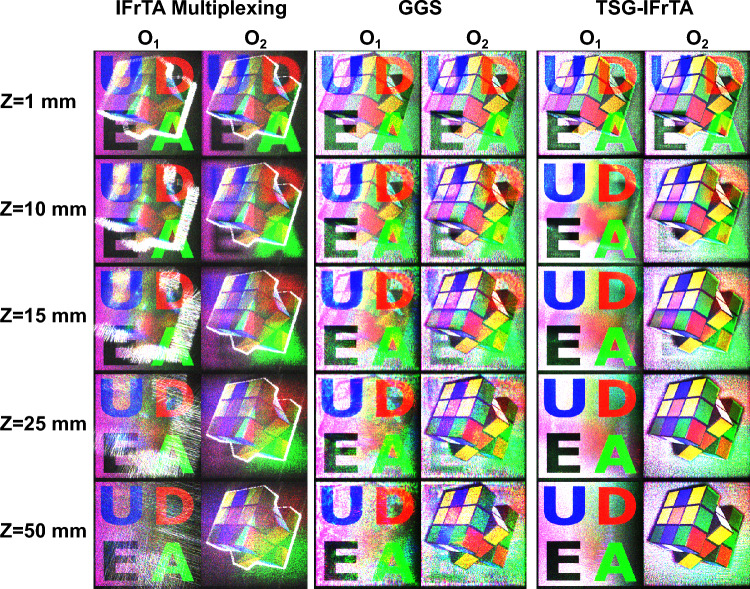
Reconstruction of holograms of the best focus plane of objects 1 and 2 when placed at different distances.

The result of this test is shown in Fig. 11. As can be seen for all distances tested, the TSG-IFrTA presents less crosstalk and increased quality. At 1 mm, the crosstalk is evident in both methods; however, at 15 mm the crosstalk is greatly reduced for the TSG-IFrTA, while remaining considerable in the GGS results. At 25 mm, the crosstalk in the TSG-IFrTA is almost eliminated, contrary to the case of the GGS results where it remains noticeable. These results show that the there is a marked dependency of the crosstalk with the distance between objects, however our proposal leads to a reduction on this effect, potentially allowing the generation of holograms from more dense scenes with closer objects along the propagation axis.

These quantitative and qualitative tests demonstrate the effectiveness of the TSG-IFrTA for multiplane hologram generation. Furthermore, thanks to the use of the C-SVD propagation, the computation time required to generate the hologram with our proposal is cut in more than half. Thus, the TSG-IFrTA offers superior quality and increased computational efficiency.

### Experimental results

The previous numerical tests highlighted the capabilities of the TSG-IFrTA. However, we now wish to complement these results with actual experimental reconstructions to demonstrate effectiveness of the TSG-IFrTA.

**Figure 12:**
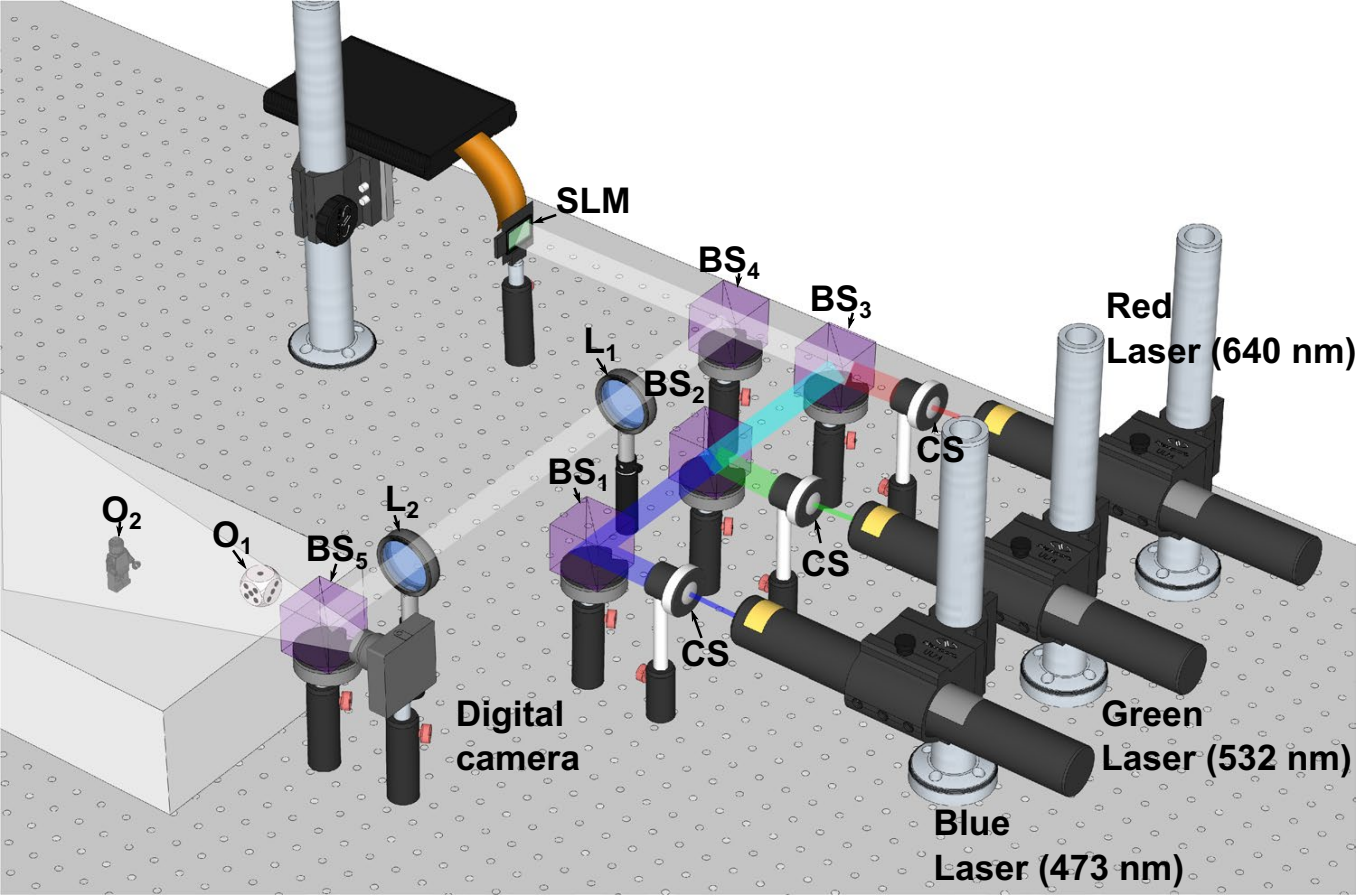
Experimental scene for the near-eye holographic augmented reality proyection system. CS: collimation system, SLM: spatial light modulator, BS: beam splitter, L: lens, O1, and O2 physical objects.

To do this, we implement an experimental setup for holographic augmented reality visualization of color multiplane scenes in a near-eye configuration, where the reconstruction can be recorded with a variable focus digital camera or observed directly with the naked eye. The full experimental setup is shown in Fig. 12.

For our experimental tests, we observed the augmented reality scene using a digital camera, and we placed two physical objects in the field of view of the camera—one at 28 cm and the other at 16 cm. Now we proceed to test the reconstruction of the scene of Fig. 1 using holograms generated with all three of the techniques discussed in this paper.

**Figure 13:**
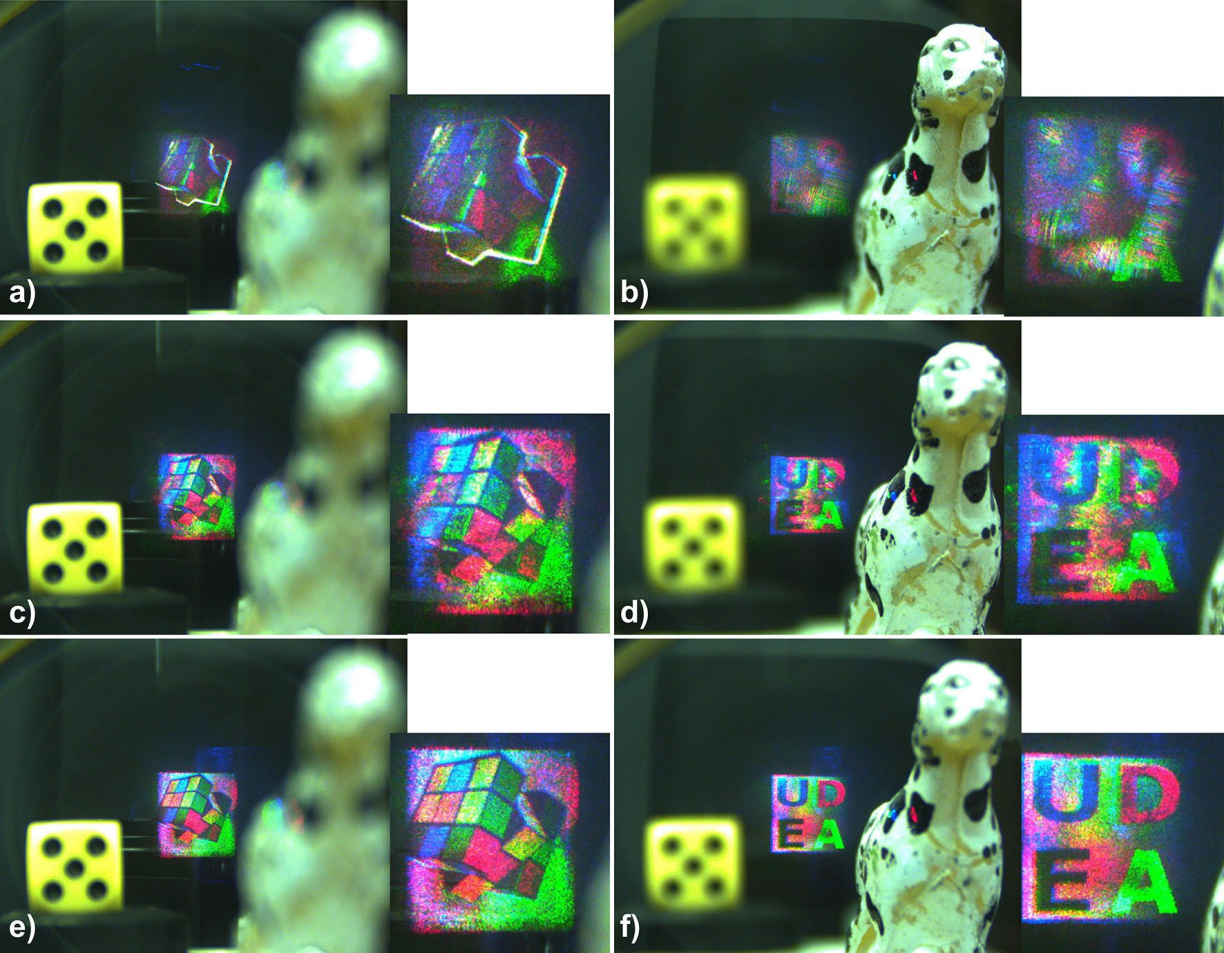
Reconstruction of (**a**) the IFrTA multiplexing hologram at 26 cm, (**b**) the IFrTA multiplexing hologram at 18 cm, (**c**) the GGS hologram at 26 cm, (**d**) the GGS hologram at 18 cm, (**e**) the TSG-IFrTA hologram at 26 cm, and (**f**) the TSG-IFrTA at 18 cm.

The images selected from the augmented reality scene can be seen in Fig. 13. Objects reconstructed from the holograms generated by each method demonstrate an excellent quantitative and visual agreement between simulations and experimental results (see Fig. 8). The way the TSG-IFrTA offers the best quality overall for the reproduction of both objects, once again without noticeable crosstalk between objects, is noticeable. Additionally, we show a scan of all focus planes between 10 and 35 cm in Visualization 4 for the IFrTA multiplexing, in Visualization 5 for the GGS, and in Visualization 6 for the TSG-IFrTA. In these videos, we can see how the scene reconstructed from the holograms is in focus at different planes from the physical objects and how the light is refocused between planes to reconstruct each virtual object.

## Discussion

The results of this paper demonstrate a full-color multiplane holographic near-eye augmented reality display capable of reproducing both 2D and 3D objects or a combination of both in a multiplane scene with excellent agreement between simulations and experimental results. The reconstructed holographic scene can be observed by either the naked eye or a camera with a variable focus objective; and it is superimposed over a real scene, providing an augmented reality experience. Our system overcomes some of the challenges that arise when attempting to reconstruct color multiplane scenes by introducing lateral and axial shifts of each plane, which ensures that color reproduction is maintained over long axial depths. We use this system to test the effectiveness of the TSG-IFrTA experimentally, demonstrating that it is a superior alternative for color multiplane hologram generation. Also, the TSG-IFrTA is capable of reproducing 2D and 3D objects at different axial positions without significant crosstalk between them, unlike the other tested approaches. Additionally, due to its superior quality, the use of TSG-IFrTA offers faster computation than other alternative algorithms.

Furthermore, our results show that the Fresnel IR can be truncated and still be used for propagation calculations with reasonable accuracy as long as the propagation distance is relatively small. This enables us to reduce the linear dependency between computation time and number of layers when holograms of 3D objects and multiplane scenes must be generated.

From the point of the hologram generation method, further research is necessary to determine the limits of the TSG-IFrTA algorithm and what other potential modifications could be introduced to the scheme detailed in this work to increase the effectiveness of multiplane hologram generation techniques. Some examples of modifications that may lead to increases in accuracy and computation speed when combined with the TSG-IFrTA are the use of mixed constraints in each plane of the target scene during hologram generation, the use of initial quadratic phases, or defining specially designed loss functions.

Regarding our implemented multiplane holographic near-eye augmented reality display, the use of fiber lasers and wave guides may enable a significant reduction in size and complexity, eventually leading to the implementation of AR glasses capable of displaying multiplane information.

## Materials and methods.

### Hologram generation parameters

For all holograms generated for this paper, the distance between the first object and the hologram plane is 18 cm, and the distance between the middle of the 3D object to the hologram plane is 26 cm. The 3D object has a total axial length of 2 cm and is divided into 20 layers with a distance between layers of 1 mm. The illumination wavelengths are 473 nm for the blue channel, 532 nm for the green channel, and 640 nm for the red channel. For all three hologram generation methods, we used 20 iterations^29^. The hologram resolution is $1920\times 1080$ pixels with a pixel size of 8 μm. The holograms were generated using parallel computing with an NVIDIA 3080 RTX GPU.

### Holographic projection scheme

For the holographic projection scheme shown in Fig. 10, we used a PLUTO-2-VIS-016 SLM with a resolution of 1920 × 1080 pixels and a pixel size of 8 μm × 8 μm. In this setup, we use three diode pumped solid-state lasers as light sources with wavelengths of 473 nm for the blue channel, 532 nm for the green channel, and 640 nm for the red channel. To enable color reconstruction with a single SLM, we use time division multiplexing, where the holograms of each color channel are projected onto the SLM in sequence and synchronized with the corresponding lasers. This technique takes advantage of the image retention capabilities of the human vision, and our setup can achieve a color scene reconstruction with a total frame rate of 20 Hz. All three lasers had output power of 150 mW. The three beams are expanded, collimated, and filtered using a spatial filter with a 40X microscope objective, a 25 µm pinhole, and a positive lens with 20 cm of focal length. The recording media is a CMOS EO-10012C camera with a pixel size of 1.6 μm × 1.6 μm and a resolution of 3840 × 2848 pixels with a Mokose variable focus 6 mm-12 mm F/2.8 lens. Only the focus adjustment was changed to vary the focus plane, leaving the aperture and tele-wide adjustment fixed.

The light reflected by the SLM passes through an optical system composed of two lenses, which enable the filtering of undesired diffraction orders from the reconstructed scenes by placing a filter on the focal plane of the first lens. This also requires multiplying the hologram of each color channels with a phase grating, to separate the modulated containing the reconstructed scene from DC term of the SLM.

By changing the focal length of these lenses, we can also control the magnification of the reconstructed scene. In our case, we use two lenses with a focal length of 10 cm. Finally, we use a beam splitter to superimpose the holographic reconstruction with a real scene, providing the augmented reality capabilities of the system.

Prior to testing the different hologram generation methods, a calibration of the system is necessary to eliminate the chromatic aberration introduced by the lenses, and to ensure that a single filter can eliminate the undesired diffraction orders from all color channels simultaneously. This is achieved by changing the phase grating period for each channel and then introducing both a lateral and axial shift for each plane of the multiplane scene during hologram generation. The value of these shifts is calibrated by generating the holograms of a 2D color calibration object at different depths and projecting it in the system. The green channel is used as reference and the shift is introduced in the red and blue channel, regenerating the hologram of each of these two channels with the new shift until optimal reconstruction of the calibration object is achieved over a large range of axial positions. Once this calibration is complete, it is not necessary to repeat it unless the lens system or filter position is changed.

### Quality metrics

To evaluate the quality of the numerically and experimentally reconstructed holograms, we used the 2D correlation coefficient (CC), defined as

5 $$CC = \frac{{\sum\nolimits_{m} {\sum\nolimits_{n} {(I_{mn} - \overline{I})(R_{mn} - \overline{R})} } }}{{\sqrt {\left( {\sum\nolimits_{m} {\sum\nolimits_{n} {(I_{mn} - \overline{I})^{2} } } } \right)\left( {\sum\nolimits_{m} {\sum\nolimits_{n} {(R_{mn} - \overline{R})^{2} } } } \right)} }}$$

where $I$ and $R$ are the intensity of the reconstructed hologram in a given plane, the reference object, $\overline{I},\,\overline{R}$ represent their mean intensity values, and $m,n$ are pixel coordinates.

The CC ranges from 1, for identical images, to 0. Compared with other metrics such as PSNR and SSIM, it is less affected by local intensity changes, which simplifies its application to experimental reconstruction of holograms.

## Supplementary Information

Supplementary Video 1.

Supplementary Video 2.

Supplementary Video 3.

Supplementary Video 4.

Supplementary Video 5.

Supplementary Video 6.

## Data Availability

The data that supports the results within this paper are available from the corresponding authors upon reasonable request.

## Code availability

The custom code and mathematical algorithms used to obtain the results within this paper are available from the corresponding authors upon reasonable request.
